# Analysing the Contact Conduction Influence on the Heat Transfer Intensity in the Rectangular Steel Bars Bundle

**DOI:** 10.3390/ma14195655

**Published:** 2021-09-28

**Authors:** Cezary Kolmasiak, Vazgen Bagdasaryan, Tomasz Wyleciał, Marek Gała

**Affiliations:** 1 Department of Production Management, Czestochowa University of Technology, Armii Krajowej 19, 42-200 Czestochowa, Poland; cezary.kolmasiak@pcz.pl (C.K.); tomasz.wylecial@pcz.pl (T.W.); 2 Institute of Civil Engineering, Warsaw University of Life Sciences—SGGW, Nowoursynowska 166, 02-787 Warsaw, Poland; 3 Institute of Electric Power Engineering, Czestochowa University of Technology, Armii Krajowej 17, 42-200 Czestochowa, Poland; marek.gala@pcz.pl

**Keywords:** rectangular bars, bar bundle, effective thermal conductivity, contact conduction, cellular medium, heat treatment

## Abstract

Bundles of steel bars, besides metal foams, are an example of cellular solids. Such bundles constitute a charge during the heat treatment of bars. The paper presents a mathematical model of transient heat transfer in a bundle of rectangular steel bars based on the energy balance method. The key element of this model is the procedure of determining the effective thermal conductivity using the electrical analogy. Different mechanisms of heat transfer occurring within the analysed medium (conduction in steel and contact conduction) are assigned corresponding thermal resistances. The discussed procedure involves expressing these resistances with the use of arithmetic relationships describing their changes in the temperature function. Thermal contact resistance has been described with the use of the relationships determined experimentally. As a result of the performed calculations, the influence of contact conduction between the adjacent bars and bundle arrangement on its heating time was established. The results of the calculations show that the heating time of bundles can be lowered by 5–40% as a result of a decrease in the thermal contact resistance. This effect depends on the bar size and bundle arrangement. From the practical point of view, the analysed problem is connected with the optimization of the heat treatment processes of steel bars.

## 1. Introduction

**Figure 1:**
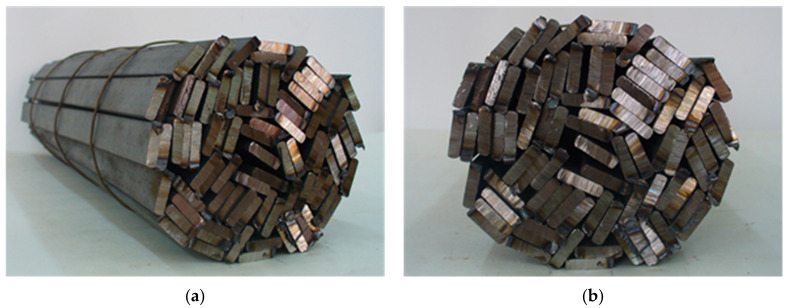
Model of the bundle of rectangular bars: (**a**) a general view, (**b**) a frontal view.

Steel bars are basic products of the steel industry. In many cases, in order to provide the required mechanical properties, bars undergo the process of heat treatment, during which they are usually heated in the form of cylindrical bundles. Bundles of bars are an example of a steel porous charge that can be encountered in industrial practice [1,2,3]. Such bundles, besides metal foams, are an example of cellular solids [4]. The present article is concerned with the problem of heating a cylindrical bundle of rectangular bars, the model of which is presented in Figure 1.

Since the length of bar bundles is repeatedly bigger than their diameter, their heating is determined by the processes of heat transfer, which occur in the radial direction. As can be seen in Figure 1, the analysed charge is characterized by the presence of spaces filled with gas and the lack of continuity of the solid phase in the radial direction. The above mentioned characteristics have a decisive influence on the process of bundle heating. The thermal energy in such a medium is transferred in a complex way as a result of: (i) conduction within the individual bars, (ii) conduction within gas, (iii) contact conduction between adjacent bars and (iv) thermal radiation between the bar surfaces [5]. The complexity of the heat transfer is the reason why a precise mathematical description of the heating process of a bundle requires using many complex dependencies. It is possible to simplify this description considerably by introducing the notion of the effective thermal conductivity *k_ef_*. This parameter is commonly used in the theory of porous [6,7,8] and nonhomogeneous [9,10,11] media. By using the effective thermal conductivity it is possible to describe a transient heat transfer within a bundle of bars [12]. This coefficient for different types of nonhomogeneous materials can be determined both with the use of experimental investigations [13] and model calculations [14].

The present article analyses the influence of contact conduction, which occurs between adjacent bars during heating of the bundle. For the quantitative description of contact conduction phenomenon the thermal contact resistance *R_ct_* has been used. The changes in this resistance in the temperature function have been established experimentally.

## 2. Materials and Methods

In the analysis of the problem it has been assumed that the charge is heated only in the radial direction, at a uniform heat flux *Q_0_* on the whole circumference. Hence, it is an axially symmetric problem. The geometry of the charge is defined by giving the outer radius of the bundle *r_0_* = 0.25 m and its length *l_b_* (it was assumed that *l_b_* = 1 m). For the purposes of the numerical solution, the area of the bundle is divided into *n* cylindrical elements with the width of (Equation (1))

(1) $$\Delta r=\frac{r_{0}}{n}$$

When carrying out the calculations, it was assumed that *n* = 100. In the middle of each element there is a node, for which a temperature is established. The radius of the *m*-th node is described with the Equation (2)

(2) $$r_{m}=r_{0}-\left(m-0.5\right)\Delta r$$

In order to solve a transient heat conduction problem, energy balance method has been used, according to which the conducted heat flux to the *m*-th element from the adjacent elements *Q_j,m_* is equal to the change in the energy content of an element during time interval Δ*τ*. Using the explicit approach, it can be represented with the Equation (3) [15]

(3) $$\rho c_{p}\varphi V_{m}\frac{\Delta t_{m}}{\Delta \tau }=\underset{j}{\sum }Q_{j,m}^{i}$$

where: *ρ*, *c_p_*—density and specific heat of steel, *φ*—bundle porosity (*φ* = 0.05), Δ*t_m_*—temperature change of the *m*-th node during the time interval Δ*τ* (Equation (4))

(4) $$\Delta t_{m}=t_{m}^{i+1}-t_{m}^{i}$$

*V_m_*—volume of the *m*-th element (Equation (5))

(5) $$V_{m}=2\pi r_{m}\Delta r$$

In the solution, it has been assumed that the specific heat of steel in the temperature function changes according to the Equation (6)

(6) $$c_{p}=0.41t+468$$

Equation (6) has been established based on literature data [16].

The heat transferred into the *m*-th element from adjacent elements amounts to (Equation (7))

(7) $$\sum_{j}Q_{j,m}^{i}=\frac{1}{R_{m-1,m}^{i}}\left(t_{m-1}^{i}-t_{m}^{i}\right)+\frac{1}{R_{m,m+1}^{i}}\left(t_{m+1}^{i}-t_{m}^{i}\right)$$

where

(8) $$R_{m-1,m}^{i}=\frac{ln\left(\frac{r_{m-1}}{r_{m}}\right)}{2\pi l_{b}k_{ef}^{i}}$$

(9) $$R_{m,m+1}^{i}=\frac{ln\left(\frac{r_{m}}{r_{m+1}}\right)}{2\pi l_{b}k_{ef}^{i}}$$

The boundary condition used in the solution is heat flux, which flows in to the charge *Q_0_*. It has two components: a convection component and a radiation component (Equation (10))

(10) $$Q_{0}^{i}=2\pi r_{0}l_{b}\left\{h^{i}\left(t_{F}^{i}-t_{0}^{i}\right)+\varepsilon \sigma \left[{\left(T_{F}^{i}\right)}^{4}-{\left(T_{0}^{i}\right)}^{4}\right]\right\}$$

where: *h*—convection heat transfer coefficient, *ε*—bundle surface emissivity (*ε* = 0.7), *σ*—Stefan–Boltzmann constant, *T_F_* and *T_0_*—thermodynamic temperatures of the furnace and bundle surface.

It was assumed that the furnace temperature is rising up to the final value as the function of time described by Equation (11)

(11) $$t_{F}=20+0.173\tau$$

where time *τ* is expressed in seconds. Upon reaching the final value, for the rest of the process temperature, *t_F_* is kept constantly at this level. Two series of calculations for the final furnace temperature equal to 750 °C and 800 °C have been performed.

Convection heat transfer coefficient *h* from Equation (10), was calculated for a given step of time *τ^i^* based on the Nusselt Nu and Rayleigh Ra numbers for the case of natural convection over horizontal cylinder (Equations (12)–(14)) [17]

(12) $$h^{i}=\frac{{\mathrm{Nu}}^{i}k_{air}^{i}}{2r_{0}}$$

(13) $${\mathrm{Nu}}^{i}={\left\{0.6+\frac{0.387{\left({\mathrm{Ra}}^{i}\right)}^{1/6}}{{\left[1+{\left(0.559/\mathrm{Pr}\right)}^{9/16}\right]}^{4/9}}\right\}}^{2}$$

(14) $${\mathrm{Ra}}^{i}=\frac{g\beta_{air}^{i}\left(t_{F}^{i}-t_{0}^{i}\right){\left(2r_{0}\right)}^{3}}{{\left(\nu_{air}^{i}\right)}^{2}}Pr$$

where: *k_air_*—thermal conductivity of air (Equation (15)), Pr—Prandtl number (for air Pr = 0.72) *g*—gravitational acceleration, *β_air_*—volume expansivity of air (Equation (16)), *ν_air_*—kinematic viscosity of air (Equation (17))

(15) $$k_{air}^{i}=0.0255+5\times 10^{-5}\left[0.5\left(t_{F}^{i}+t_{0}^{i}\right)\right]$$

(16) $$\beta_{air}^{i}=\frac{1}{273+\left[0.5\left(t_{F}^{i}+t_{0}^{i}\right)\right]}$$

(17) $$\nu_{air}^{i}=\left\{13.3+0.108\left[0.5\left(t_{F}^{i}+t_{0}^{i}\right)\right]\right\}\times 10^{-6}$$

Equations (15) and (17) were determined through approximation of the tabular data [18].

In order to calculate thermal resistances described by the Equations (8) and (9), it is necessary to know the effective thermal conductivity *k_ef_* of the heated charge. The value of this parameter has been calculated for a current temperature of a given element *t_m_^i^* in a separate procedure. For this purpose, the concept of an elementary cell has been used. It is a commonly used approach in which the effective thermal conductivity is calculated based on the analysis of thermal resistances (the so-called electrical analogy) [19,20,21,22,23]. The coefficient *k_ef_* for a defined elementary cell has been calculated from the Equation (18)

(18) $$k_{ef}=\frac{l_{cell}}{R_{cell}}$$

where: *l_cell_*—characteristic dimension of the elementary cell, *R_cell_*—total thermal resistance of the cell. The values of *l_cell_* parameter, which are characteristic for the analysed computational cases of the charge, are presented in Table 1.

It has been assumed that the thermal resistance of the cell is the sum of conduction resistance in a layer of steel *R_st_* and thermal contact resistance between the adjacent layers of bars *R_ct_* (Equation (19))

(19) $$R_{cell}=R_{st}+R_{ct}=\frac{l_{cell}}{k_{st}}+R_{ct}$$

**Figure 2:**
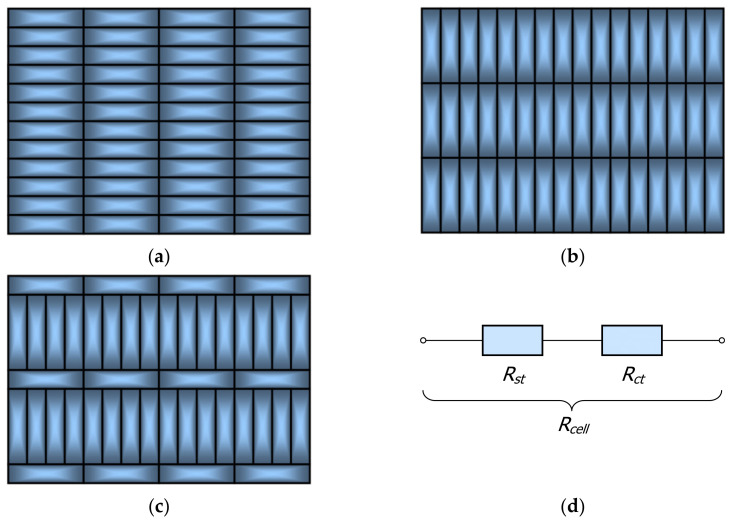
Samples of beds of rectangular bars with a horizontal (**a**), vertical (**b**) and mixed (**c**) arrangement, (**d**) the scheme of the resistances connection used to calculate total thermal resistance *R_cell_*.

The scheme of the resistances connection used to calculate the total thermal resistance *R_cell_* is presented in Figure 2d.

It has been assumed that the material of the bars is low-carbon steel S 235JRH [24]. Changes in thermal conductivity of this kind of steel in the temperature function are described by Equation (20) [23]. The unit of the *k_st_* coefficient described by Equation (20) is W/(m·K).

(20) $$k_{st}=1.2\times 10^{-8}t^{3}-3.2\times 10^{-5}t^{2}-1.2\times 10^{-2}\;t+51.3$$

The considered model takes into account the fact that the resistance *R_ct_* changes in the temperature function. The dependencies that describe it have been established on the basis of experimental research with the use of a guarded hot plate apparatus [25,26]. The way of determining this resistance has been described in detail in [27]. Due to the adopted research methodology, the *R_ct_* resistance also takes into account thermal radiation occurring between the surfaces of the bars. This fact is worth emphasizing because it has been shown that in the temperature range of 25–800 °C, approximately 30% of heat within the bar bundle is transferred through radiation [28]. The tested samples were packed beds of rectangular bars in horizontal, vertical and mixed arrangements, which have been schematically presented in Figure 2.

**Figure 3:**
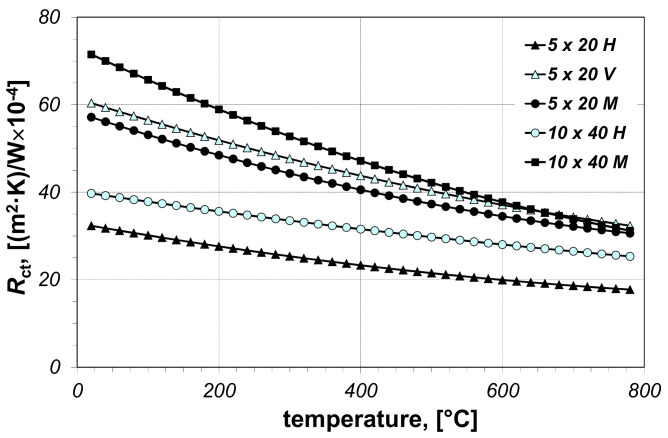
Measured values of the thermal contact resistance *R_ct_* of the beds of rectangular bars: H—horizontal sample, V—vertical sample, M—mixed sample.

The measurements have been performed for five samples in total—three of them from 5 × 20 mm bars (in the horizontal, vertical and mixed arrangement), and two from 10 × 40 mm bars (in the horizontal and mixed arrangement). The measurement results of thermal contact resistance are presented in Figure 3. As can be seen, this parameter of all investigated samples decreases with the increase in temperature. Its changes with respect to temperature were approximated by the Equation (21)

(21) $$R_{ct}=\left(A_{1}t^{2}+A_{2}t+A_{3}\right)\times 10^{-4}$$

The values of the *A_1_–A_3_* coefficients from this polynomial corresponding to the particular samples are summarized in Table 1, which also presents the values of *l_cell_* parameter.

Samples 5 × 20 H and 10 × 40 M have the smallest and highest values of the resistance *R_ct_*, respectively. Therefore, it was assumed that the thermal contact resistance in the bundles of steel rectangular bars can assume values between these two extreme cases. Consequently, the effective thermal conductivity calculations were made for the two values of the thermal contact resistance (minimum *R_ct-min_* and maximum *R_ct-max_*). The changes in the resistances *R_ct-min_* and *R_ct-max_*, in relation to temperature, are described by the Equations (22) and (23) respectively

(22) $$R_{ct-min}=\left(1.2\times 10^{-5}t^{2}-0.0288\;t+32.91\right)\times 10^{-4}$$

(23) $$R_{ct-max}=\left(2.9\times 10^{-5}t^{2}-0.0762\;t+72.86\right)\times 10^{-4}$$

Using the Equations (22) and (23), two limiting values of coefficient *k_ef_* can be obtained. The effective thermal conductivity of a given bundle of rectangular bars should remain between these values. This approach appears to be reasonable because the *k_ef_* coefficient is not a material characteristic, unlike the thermal conductivity of solids, but expresses only the ability of a given medium (the bar bundle in this example) to transfer heat. For the considered charge, this ability is largely dependent on the conditions for heat conduction through the interfaces of adjacent bars in the bundle, which may vary between individual bundles.

## 3. Results and Discussion

**Figure 4:**
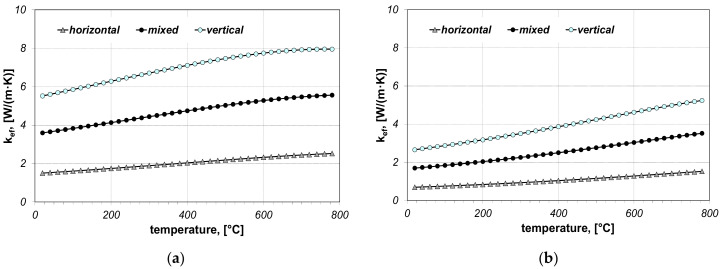
Calculated values of the coefficient *k_ef_* for the bundle of 5 × 20 mm bars: (**a**) results obtained for *R_ct-min_*, (**b**) results obtained for *R_ct-max_*.

**Figure 5:**
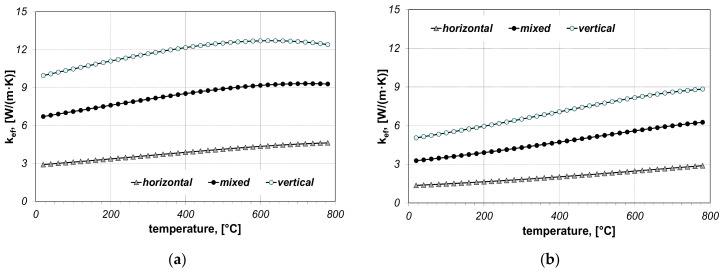
Calculated values of the coefficient *k_ef_* for the bundle of 10 × 40 mm bars: (**a**) results obtained for *R_ct-min_*, (**b**) results obtained for *R_ct-max_*.

When performing the calculations, twelve cases in total were taken into account and they concerned: two bar sizes (5 × 20 mm, 10 × 40 mm), three bar arrangements (horizontal, vertical and mixed) and two values of contact resistance (*R_ct-min_* and *R_ct-max_*). The results of calculations of the effective thermal conductivity *k_ef_* are discussed in first. Values of this parameter are shown in Figure 4 and Figure 5.

The coefficient *k_ef_* is predominantly growing in the temperature function and, for the analysed bundles, it is in the range from 1.5 to 12.7 W/(m·K), while thermal conductivity of bars *k_st_* within the analysed temperature range is decreasing from 51 to 28 W/(m·K). This shows how significantly the cellular structure of the bar bundle influences its ability to transfer heat. The value of *k_ef_* depends on the bundle arrangement, bar size and thermal contact resistance. The obtained results show that the bundle arrangement has the biggest influence on the changes in *k_ef_*, which results from the change in the cell dimension *l_cell_*.

**Figure 6:**
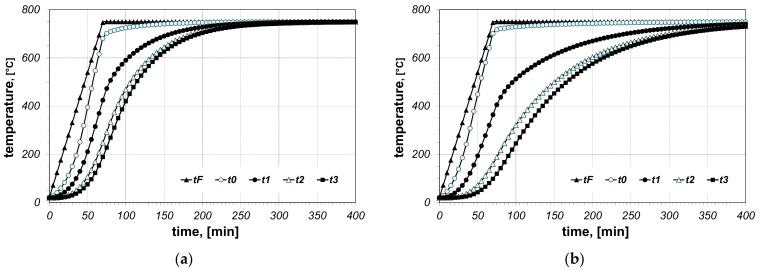
Calculated values of temperatures for heating of a 5 × 20 bar bundle with horizontal arrangement: (**a**) results obtained for *R_ct-min_*, (**b**) results obtained for *R_ct-max_*.

**Figure 7:**
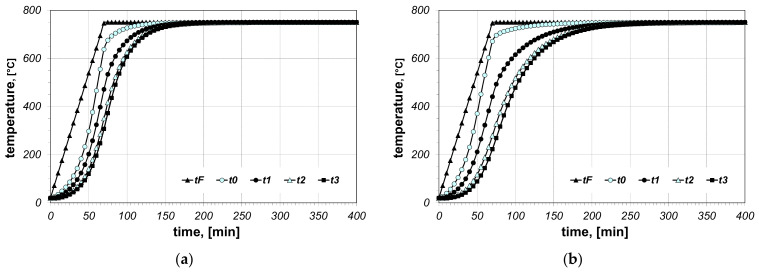
Calculated values of temperatures for heating of a 5 × 20 bar bundle with mixed arrangement: (**a**) results obtained for *R_ct-min_*, (**b**) results obtained for *R_ct-max_*.

**Figure 8:**
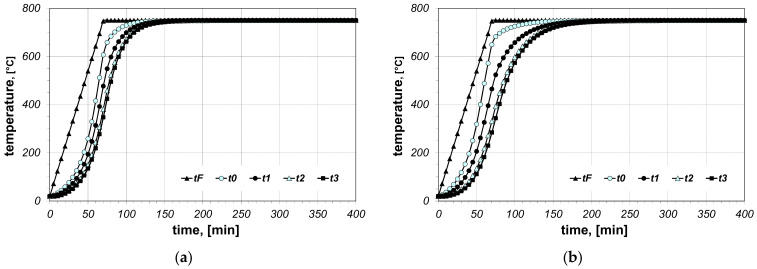
Calculated values of temperatures for heating of a 5 × 20 bar bundle with vertical arrangement: (**a**) results obtained for *R_ct-min_*, (**b**) results obtained for *R_ct-max_*.

**Figure 9:**
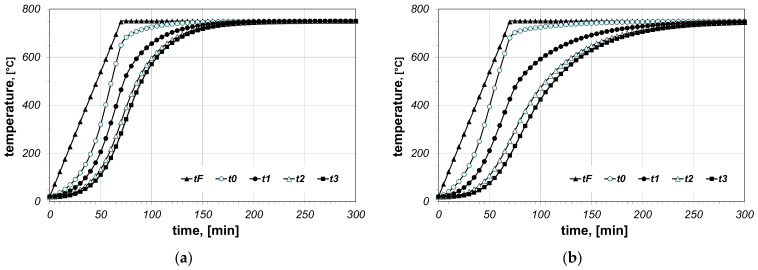
Calculated values of temperatures for heating of a 10 × 40 bar bundle with horizontal arrangement: (**a**) results obtained for *R_ct-min_*, (**b**) results obtained for *R_ct-max_*.

**Figure 10:**
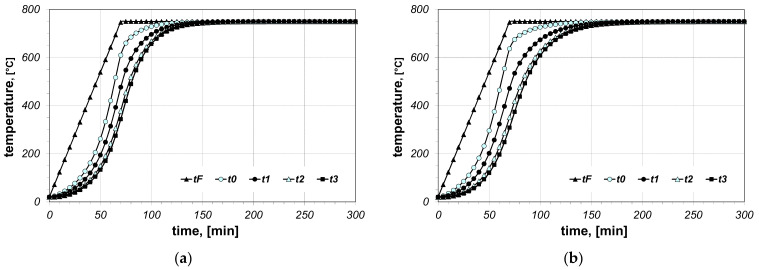
Calculated values of temperatures for heating of a 10 × 40 bar bundle with mixed arrangement: (**a**) results obtained for *R_ct-min_*, (**b**) results obtained for *R_ct-max_*.

**Figure 11:**
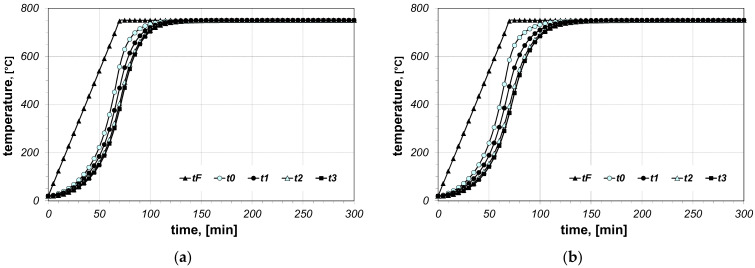
Calculated values of temperatures for heating of a 10 × 40 bar bundle with vertical arrangement: (**a**) results obtained for *R_ct-min_*, (**b**) results obtained for *R_ct-max_*.

The results of the calculations of bundle heating obtained for the final furnace temperature equal to 750 °C, for chosen cases (type of arrangement and bar size), are presented in Figure 6, Figure 7, Figure 8, Figure 9, Figure 10 and Figure 11. The diagrams present the changes in furnace temperature *t_F_*, as well as four chosen points in the section of the bundle, in the time function. The marked points relate to the following locations of the charge: *t_0_*—*r_0_* (the surface of the charge), *t_1_*—2/3 *r_0_*, *t_2_*—1/3 *r_0_*, *t_3_*—the axis of the charge. Each picture presents results for a sample with identical geometry, although differing in the value of thermal contact resistance. Diagrams marked with the letter (a) relate to resistance *R_ct-min_*; diagrams marked with the letter (b) relate to resistance *R_ct-max_*. Therefore, comparing both diagrams from one figure shows us the difference in the course of heating depending on the value of *R_ct_* resistance assumed in the model. As can be seen, the difference between the *R_ct-max_* and *R_ct-min_* values is changing in the temperature function.

For the further analysis, the percentage difference of resistance Δ*R_ct_* was calculated (Equation (24))

(24) $$\Delta R_{ct}\left(t\right)=\frac{R_{ct-max}\left(t\right)-R_{ct-min}\left(t\right)}{R_{ct-max}\left(t\right)}\times 100\%$$

Within the analysed temperature range, this parameter drops from 54.8% to 43.2%, with the average value of 50%. These changes can be described with the following polynomial (Equation (25))

(25) $$\Delta R_{ct}\left(t\right)=1.13\times 10^{-5}t^{2}-6.1\times 10^{-3}t+54.8$$

Therefore, the difference in the value of thermal contact resistance *R_ct_* between the extreme cases within the whole temperature range is relatively high.

While analysing the heating of bar bundles, attention was paid to the time which is necessary to achieve the temperature of 720 °C in their axes (this point corresponds to temperature *t_3_*). The times obtained for particular cases are collated in Table 2. The values from the second column denoted by the *τ_min_* symbol relate to bundles characterized by thermal contact resistance *R_ct-min_*. The values from the third column (*τ_max_*) relate to bundles characterised by *R_ct-max_* resistance. The last column presents the percentage difference of both times, which has been defined by the Equation (26)

(26) $$\Delta \tau =\frac{\tau_{max}-\tau_{min}}{\tau_{max}}$$

From the comparison of the Δ*τ* parameter, it shows that the influence of the thermal contact resistance on the heating time grows bigger when the effective thermal conductivity decreases. The *k_ef_* coefficient in the considered model, along with the *R_ct_* resistance, is also a function of the characteristic dimension of the elementary cell. The bigger the value of *l_cell_*, the bigger the value of *k_ef_*. Therefore, the biggest time reduction is obtained in bundles in which the bars are arranged circumferentially (a small value of *l_cell_*). The smallest reduction relates to the bundles in which bars are arranged radially (the biggest value of *l_cell_*). However, the above mentioned arrangements of bars are idealized as they are difficult to realize in industrial conditions. The real bundles of bars, as presented in the model in Figure 1, are characterized by an irregular arrangement, which is similar to a mixed arrangement. The data in Table 2 show that for a charge with such a geometry reduction in the thermal contact resistance within the analysed limits can lead to a shortening in the heating time at the level of approximately 10 to 30%. At the same time, the observed reduction becomes bigger with the decreasing bar dimensions.

Table 3 presents analogical results; however, this time the results have been obtained for the final furnace temperature equal to 800 °C. For this case, there is no heating diagram. Since the higher furnace temperature contributes to the intensification of heat transfer, times *τ_min_* and *τ_max_* are correspondingly shorter in this situation. The percentage reduction of the heating time Δ*τ* is also lower in this situation. However, the reduction of Δ*τ* in comparison with the previous case does not exceed 3%.

## 4. Conclusions

On the basis of the model calculations, it has been shown that reducing the thermal contact resistance in a bundle of rectangular steel bars can decrease the heating time of such a charge from 5% to 40%. Thermal contact resistance can be reduced by a careful bar arrangement, which consists of avoiding the crossing of bars. Another factor that contributes to this phenomenon is using adequately increased forces while preparing the bundles. However, the precise description of this problem from a quantitative point of view requires further investigation and will be the next stage of works realized by the authors in this area.

## Figures and Tables

**Table 1:** Values of the coefficients *A_1_–A_3_* from Equation (21) and characteristic dimension of the elementary cell *l_cell_* obtained for particular samples.

*Sample*	*A_1_*	*A_2_*	*A_3_*	*l_cell_*, m
5 × 20 H	1.2 × 10^−5^	−0.0288	32.91	0.0050
5 × 20 V	1.8 × 10^−5^	−0.0513	61.41	0.0200
5 × 20 M	2.3 × 10^−5^	−0.0533	58.16	0.0125
10 × 40 H	6.7 × 10^−6^	−0.0243	40.21	0.0100
10 × 40 M	2.9 × 10^−5^	−0.0762	72.86	0.0250

**Table 2:** Heating times of individual samples in a furnace with the temperature of 750 °C.

*Sample*	*τ_min_*, min	*τ_max_*, min	Δ*τ*, %
5 × 20 H	222	365	39.2
5 × 20 V	139	192	27.6
5 × 20 M	120	151	20.5
10 × 40 H	152	220	30.9
10 × 40 M	121	138	12.3

**Table 3:** Heating times of individual samples in a furnace with the temperature of 800 °C.

*Sample*	*τ_min_*, min	*τ_max_*, min	Δ*τ*, %
5 × 20 H	177	280	36.8
5 × 20 V	117	155	24.5
5 × 20 M	103	126	18.3
10 × 40 H	125	175	28.6
10 × 40 M	105	116	9.5

## Data Availability

Not applicable.

## References

[1] Kolmasiak C., Wyleciał T. (2018). Heat treatment of steel products as an example of transport phenomenon in porous media. Metalurgija.

[2] Musiał M. (2013). Numerical Analysis of the Process of Heating of a Bed of Steel Bars. Arch. Metall. Mater..

[3] Wyczółkowski R., Urbaniak D. (2016). Modeling of Radiation in Bar Bundles Using the Thermal Resistance Concept. J. Thermophys. Heat Transf..

[4] Wyczółkowski R., Bagdasaryan V., Szwaja S. (2021). On Determination of the Effective Thermal Conductivity of a Bundle of Steel Bars Using the Krischer Model and Considering Thermal Radiation. Materials.

[5] Wyczółkowski R., Gała M., Szwaja S., Piotrowski A. (2021). Determination of the Radiation Exchange Factor in the Bundle of Steel Round Bars. Energies.

[6] Kaviany M. (1995). Principles of Heat Transfer in Porous Media.

[7] Van Antwerpen W., du Toit C.G., Rousseau P.G.A. (2010). Review of Correlations to Model the Packing Structure and Effective Thermal Conductivity in Packed Beds of Mono-Sized Spherical Particles. Nucl. Engine Des..

[8] Wyczółkowski R., Benduch A. (2014). The experimental study of the effective thermal conductivity of bundles of rectangular steel sections. CEER.

[9] Kula D., Wodzyński Ł. (2020). Transfer of thermal fluctuations through the building partition formed by periodic composite material. Acta Sci. Pol. Arch..

[10] Wągrowska M., Szlachetka O. (2016). Distribution of temperature in multicomponent functionally graded multilayered composites. Acta Sci. Pol. Arch..

[11] Wozniak C., Wagrowska M., Szlachetka O. (2015). On the tolerance modelling of heat conduction in functionally graded laminated media. J. Appl. Mech. Tech. Phys..

[12] Sahay S.S., Krishnan K. (2007). Model Based Optimization of Continuous Annealing Operation for Bundle of Packed Rods. Ironmak. Steelmak..

[13] Dutkowski K., Kruzel M. (2021). Experimental Investigation of the Apparent Thermal Conductivity of Microencapsulated Phase-Change-Material Slurry at the Phase-Transition Temperature. Materials.

[14] Du Y., Ge Y. (2021). Multiphase Model for Predicting the Thermal Conductivity of Cement Paste and Its Applications. Materials.

[15] Cengel Y.A. (2002). Numerical Methods in Heat Conduction. Heat Transfer and Mass Transfer—A Practical Approach.

[16] Malinowski Z. (2005). Numerical Modeling in Plastic Processing and Heat Transfer.

[17] Cengel Y.A. (2002). Natural Convection. Heat Transfer and Mass Transfer—A Practical Approach.

[18] Cengel Y.A. (2002). Table A-15 Properties of air at 1 atm pressure. Heat Transfer and Mass Transfer—A Practical Approach.

[19] Coquard R., Rochais D., Baillis D. (2012). Conductive and Radiative Heat Transfer in Ceramic and Metal Foams at Fire Temperatures. Fire Technol..

[20] Trevisan S., Wang W., Laumert B. (2021). Coatings utilization to modify the effective properties of high temperature packed bed thermal energy storage. Appl. Ther. Eng..

[21] Wyczółkowski R., Gała M., Bagdasaryan V. (2020). Model of complex heat transfer in the package of steel rectangular steel sections. Appl. Sci..

[22] Wyczółkowski R. (2016). Computational model of complex heat flow in the area of steel rectangular section. Proc. Eng..

[23] Öchsner A., Murcg G.E., de Lemos M.J.S. (2008). Cellular and Porous Materials: Thermal Properties Simulation and Prediction.

[24] European Steel and Alloy Grades/Numbers Steel Number. http://www.steelnumber.com/en/steel_composition_eu.php?name_id=645.

[25] (2012). Standard Practice for Using a Guarded-Hot-Plate Apparatus or Thin-Heater Apparatus in the Single-Sided Mode.

[26] (2013). Standard Test Method for Steady-State Heat Flux Measurements and Thermal Transmissions Properties by Means of Guarded-Hot-Plate Apparatus.

[27] Wyczolkowski R., Radomiak H., Wylecial T. Computational model of effective thermal conductivity of the steel section bundle. Proceedings of the 10th International Conference on Computational Heat Mass and Momentum Transfer (ICCHM2T 2017).

[28] Wyczółkowski R., Boryca J. (2019). Analysis of Thermal Radiation in the Heating of Steel Round Bar Bundles. Acta Phys. Pol. A.

